# From origins to nomenclature: illuminating the landscape of CNS fibroblasts and fibroblast-like cells

**DOI:** 10.1186/s40478-026-02236-8

**Published:** 2026-01-30

**Authors:** Jianing Lin, Kuo Liao, Sebastian A. Lewandowski

**Affiliations:** Department of Clinical Neuroscience, Centre for Molecular Medicine, Karolinska Institutet, Karolinska Hospital, Stockholm, Sweden

**Keywords:** Central nervous system, Fibroblasts, Perivascular fibroblasts, Vascular leptomeningeal cells, Type A pericytes

## Abstract

Fibroblasts are a group of stromal cells that contribute to the scarring process in many neurological conditions in the central nervous system (CNS). Recently, single-cell sequencing efforts allowed an in-depth understanding of their cell origins and subpopulation profiles. Meanwhile, vascular leptomeningeal cells and the “type A pericytes" were also proposed as CNS fibroblast-like cells in the last decade by histological, functional and transcriptomic analysis. While these cells share overlapping features with CNS fibroblasts, the inconsistent use of nomenclature and partially overlapping cell-type markers is likely to cause confusion within the growing field of neurobiology. In this review, we will delineate the current knowledge of subtypes and functions of CNS fibroblasts, with special focus on the source of PVFs during development and the nomenclature origins of other similar cell types. We aim to provide comprehensive insights into these cells with similar functions or transcriptomic profiles.

## Introduction

Fibroblasts are best-known for their roles in forming and remodeling extracellular matrix (ECM), which leads to a normal healing process or abnormal fibrosis in a wild range of diseases in many organs [1]. CNS fibroblasts are found not only in the perivascular space and meninges of the brain and spinal cord, but also in the choroid plexus and meninges surrounding the optic nerve [2, 3]. CNS fibroblasts serve versatile roles in physiological or disease conditions. They become highly proliferative and synthesize matrix proteins in the context of injury or inflammation, which maintains tissue integrity in the lesions, but at the same time, creates an inhibiting environment for remyelination and neuronal growth [4]. Both meningeal fibroblasts (MFs) and perivascular fibroblasts (PVFs) contribute to fibrosis in penetrating injuries such as transected spinal cord injury (SCI) that tears apart the meninges [5]. Meanwhile, the vessel-related PVFs gain more attention in response to non-penetrating injuries such as contusive-compressive SCI, stroke and neuroinflammation, in which the meninges remain intact [6–8]. CNS fibroblasts also closely interact with immune cells, which is highlighted by their crosstalk with infiltrating T cells during CNS fibrotic scarring via the IFN-γ signaling pathway, and their interface with meningeal B cells during development through CXCL12-CXCR4 axis affecting immune tolerance [8, 9]. They play indispensable roles in neurovascular development and contribute to CNS homeostasis.

Advanced sequencing techniques have made it possible to identify cell types based on a set of annotated marker genes, even for low abundance cells like the CNS fibroblasts [10, 11]. We now have a better understanding of fibroblast heterogeneity, as multiple studies have further identified distinct subpopulations based on their transcriptomic profiles [11]. An early single-cell RNA sequencing study identified *Pdgfra* positive vascular leptomeningeal cells (VLMCs) from other oligodendrocyte lineage cells, which is a distinct population in the leptomeninges, the delicate arachnoid and pia mater covering the brain and spinal cord [12]. These VLMCs were later identified as fibroblast-like cells but their functions were less documented [13, 14]. While fibroblasts are generally regarded as the main source of CNS fibrosis, a group of “type A pericytes” have also been observed to be activated across a range of CNS lesions [15, 16]. Although “type A pericytes” display biological behaviors similar to those of vessel-derived PVFs, anatomical and transcriptomic analyses suggest they potentially form a distinct cell population, and their functions outside of fibrotic scarring remain largely unknown.

**Fig. 1:**
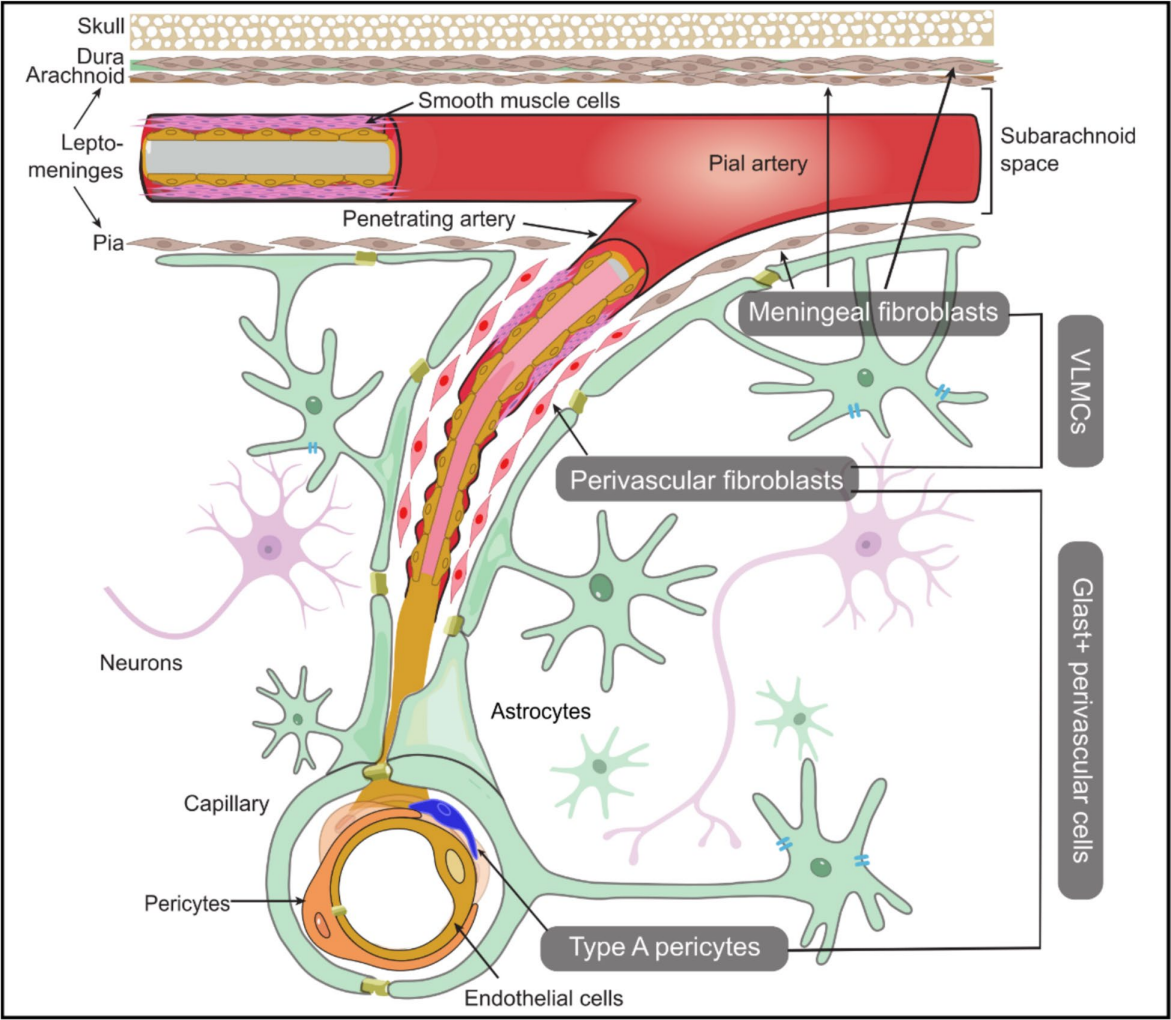
Fibroblasts, VLMCs and Glast+ perivascular cells in the CNS. CNS fibroblasts can be classified based on their anatomical locations in the meninges, perivascular space and choroid plexus. Meningeal fibroblasts can be found in the dural, arachnoid and pial mater, while the pial fibroblasts and perivascular fibroblasts are closely related in the CNS development and share very similar transcriptomic profiles. The term VLMCs was introduced only in some single-cell studies where cell clusters were found related to the vessels and meninges. They were recognized as fibroblasts based on the overlapping expression profiles, although their functions have been less characterized. *Glast/Slc1a3* expression labels a population of perivascular cells exhibiting fibrotic characteristics during CNS injury, mainly comprising perivascular fibroblasts and a subset of pericytes. Schematic representation, cells are not drawn to scale

The inconsistent nomenclatures of these fibroblasts or fibroblast-like cells in CNS vasculature across literatures are likely to cause confusion within the communities of neurobiologists studying CNS cell type populations. Therefore, this review aims to update the current understanding of cellular origin of PVFs during CNS development, and more importantly, to conclude existing knowledge of CNS fibroblasts subtypes and functions based on the advancing transcriptomic analysis. More importantly, we will clarify the nomenclatural origins of type-A pericytes and VLMCs, and highlight their definitions and markers (Fig. 1). The elucidation of features and nomenclature origins of these matrix-producing cells in the CNS will not only improve our understanding of the delicate fibrotic process, but also resolve current ambiguities and standardize their classification within the field.

## Emerging focus on the CNS fibroblasts and the perivascular space in the CNS

The study of CNS fibroblasts originated from the studies on fibrosis back in the twentieth century, when many researchers discovered the formation of scar tissue in CNS lesions (reviewed by Fehlberg and Lee) [17]. Opinions were divided on the main cellular sources of tissue scarring in CNS lesions. In early studies, astrocytes were found activated following injury as the main responders, generating proteoglycans that form the glial scar. This process restricted the spread of neuroinflammation but simultaneously inhibited axon regeneration [18–20]. Some studies highlighted the involvement of MFs, especially in penetrating CNS lesions with torn meninges. It was hypothesized that MFs migrate into the injury site in the acute phase and proliferate, but their distribution was strictly controlled by astrocytic ephrin-B2, resulting in their subsequent segregation from the glial scar [21]. It is also noteworthy that a study reported a group of NG2 + cells with a unique retinoic acid expression profile migrating from the adjacent arachnoid mater into the injury site in a spinal cord contusion model [22]. Although without validation of fibroblast-specific markers, the NG2 + cells described are likely MFs given the arachnoid location and similar retinoic acid synthesis enzyme expression [22, 23]. Efforts in keeping the dura continuity by applying synthetic dura sheaths, dura mater repair or allograft replacement alleviated fibroblast invasion and formation of connective tissues in the spinal cord [24–27].

On the other hand, in the non-penetrating neurological conditions such as ischemic stroke, intracerebral hemorrhage and multiple sclerosis, proliferating fibroblasts from the parenchyma were believed to be responsible for the formation of fibrotic scarring in lesions when the meninges remained intact [28–30]. However, there were a lack of specific lineage tracing tools to distinguish PVFs and MFs, making it challenging to characterize their relative contribution.A recent study applied transcranial delivery of 4-OH-tamoxifen on *Col1a2*-CreER^T2^ mice to sparsely label dural fibroblasts but not perivascular or leptomeningeal fibroblasts and revealed the recombined dural fibroblasts in the lesion site in a mouse stroke model [31]. In the future, proper lineage tracing tools such as the *Crabp2*-CreER transgenic mouse line that specifically labels meningeal fibroblasts can be useful in studying functions and behaviors of fibroblast subclusters in more neurological disorders [32].

Nonetheless, these matrix-producing cells were once either termed as “CNS-resident stromal cells” or “perivascular stromal cells” [30, 33]. They are collectively termed PVFs based on their anatomical location in the perivascular spaces (PVS) surrounding blood vessels within the CNS parenchyma. The first description of PVS was much earlier than the understanding of PVFs in the brain, followed by the subsequent discovery of perforating vessels-related places by Virchow and Robin in 1850s [34, 35]. There were ongoing debates in the last century regarding the anatomical connection between the subarachnoid space and PVS, and an electron microscopic study in 1991 uncovered a continuous and complete pial sheath around the subpial and the penetrating arteries into brain parenchyma [35, 36]. This study confirmed the direct anatomical continuity of the perivascular compartment around intracerebral vessels and pial arterioles, rather than being directly connected to the subarachnoid space, and further revealed that the pial sheath becomes increasingly perforated in smaller arterioles while being nearly absent around veins [36]. In human, pial sheath patterns of penetrating arteries are distinct across different brain regions, with two leptomeningeal layers for the basal arteries and one leptomeningeal layer for cortical arteries [37]. One mouse study further characterized that pia forms one or more layers around leptomeningeal and penetrating arterioles on the brain surface with heterogeneous patterns, which don’t correlate with brain regions or vessel sizes, highlighting the need to better understand cross-species relevance with further investigations [38]. With the in-depth understanding of brain vasculature, PVS is now widely defined as the compartment surrounding the vascular wall, delimited by the astrocytic end-foot layer. In addition to the PVF cells, PVS hosts resident macrophages and infiltrating lymphocytes and collectively enables immune surveillance and neuroinflammatory responses, which positions PVS as a critical hub for both homeostasis and pathological processes in the CNS [39].

## Cellular origins of fibroblasts in the developing CNS

While most studies focused on the behaviors of PVFs during injuries, their cellular origins and biological events in CNS development initially received less attention. Investigations into the structural parallels of pial and penetrating arterioles have provided several important clues. Initially, the researchers found similar ultrastructure of desmosomes and small nexus junctions in the pial sheath and the sheath around intracerebral vessels [36]. By using specific markers targeting different laminin isoforms and other ECM molecules, one study visualized the perivascular compartments of the whole vascular network in the brain parenchyma. The penetrating arterioles are ensheathed by a laminin α1/plectin positive layer, which is the same as the layer in pia but different from that found in capillaries [40]. In the postnatal mouse brain, *Col1a1* positive fibroblasts identified by targeted antibody are mostly located near the meninges at postnatal day 7, and the population expands dramatically away from the pial surface and enters the parenchymal PVS in all brain regions during postnatal day 7 (P7) and P21 [41]. However, the antibody-based detection of Col1a1 mainly labels the extracellular collagen and the whole membrane region, which is less cell-specific. Nonetheless, these studies clearly inferred the perivascular sheath in the brain parenchyma as the continuation of the pia, suggesting possible relations of pial fibroblasts and PVFs.

Implementation of *Col1a1* promoter-driven transgenic mice greatly aided visualization of CNS fibroblasts, where the MFs can be easily distinguished from PVFs based on their anatomical locations [42, 43]. In general, PVFs are observed in penetrating arterioles, arteriole-capillary transition zones, and some large venules in cerebral cortex, which makes them distinct from other mural cells [43]. It is important to note that PVFs are absent in vasculature in retina, which can be explained by the fact that the pia sheath only ensheathed the optic nerve but not the retina vessels, supporting the notion of pial origin of PVFs [43]. From a transcriptomic point of view, the maturation of fibroblast populations during brain development between embryonic day 12 (E12) and E14 follows a ventral-to-dorsal pattern, and MFs can be clustered based on their differentiated gene expression profiles referring to distinct meningeal layers [11, 44]. During the period, two clusters of proliferating MFs were characterized by the expression of cell cycle genes, and MFs showed an active retinoic acid synthesis profile that is indispensable for brain development [44, 45]. One distinct pial cluster with elevated μ-crystallin expression was identified in the dorsal thalamus and the choroid plexus, but their specific functions remain unclear [44]. Another study also profiled MFs development in the embryonic stage, uncovering detailed transcriptomic transition from E7 to E18 when primitive fibroblast-like cells give rise to *Col15a1*-expressing pia and *Slc22a2*-expressing arachnoid fibroblasts [46]. Upon the establishment of fibroblast network in the meninges at around E14 to E15, the MFs were initially restricted to meningeal vessels until postnatal day 5 (P5). Between P5 and P15, fibroblasts begin to migrate out of meninges and into the brain parenchyma. As they proliferate, these cells progressively envelop penetrating arterioles, eventually forming a PVFs layer around these blood vessels [47]. One current study filled the gap in understanding functional human meningeal development from post- conception week 8 to week 20. In general, leptomeningeal fibroblasts display a transitional expression profile, from proliferation and differentiation at the early fetal stage, to cell polarity development and vessel formation at the early mid-fetal stage, to a relative mature state with higher adhesion marker expression and more RNA splicing events at the late mid-fetal stage [48]. This elaborate development highlights dynamic embryonic and postnatal expansion of brain fibroblasts and their coordinated development with the cerebral vasculature.

## CNS fibroblast subtypes and their proposed functions

The advent of next-generation sequencing has enabled a clearer understanding of the differences among CNS fibroblasts at the molecular level. In the mouse brain, the transcriptomic profiles of fibroblasts were precisely characterized based on the anatomical meningeal layers [11]. In the study, the parenchymal PVFs (termed brain fibroblast 1a, BFB1a) as well as pial fibroblasts and pial PVFs (together termed as BFB1b) share consistent *Lama1* expression, but the BFB1a cluster shows much abundant expression of fibril-associated collagen genes, including *Col15a1*, *Col12a1*, and basement membrane collagens *Col4a1* and *Col4a2 *[11]. On the other hand, pial fibroblasts express higher levels of *Mgp* and *Serpine2*, which are indirectly related to the ECM remodeling and regulation [49, 50]. The difference between the PVFs and pial fibroblasts was also highlighted in a histology analysis that revealed significantly lower expression of nestin and vimentin in vessel-related PVFs, despite their developmental association and the common markers PDGFR-β and COL1A1 [51]. Additional fibroblast clusters were also identified in arachnoid, dura, choroid plexus and median eminence with distinctive markers, allowing a thorough understanding of CNS fibroblasts on an anatomical basis [11]. Compared with mouse dura, human dural fibroblasts display more complicated and heterogeneous profiles, each characterized by proliferation, activation or ECM organization with immune regulation markers [52]. Brain fibroblasts have region-specific distribution and expression patterns. In contrast to the parenchymal PVFs residing on the vessels with larger diameter in the cortex, fibroblasts were found adjacent to the capillaries in the median eminence [53]. Fibroblasts in the median eminence and pituitary gland show expression profiles closer to the fibroblasts in the choroid plexus, which is related to the higher vascular permeability in these regions [53, 54]. These molecular and functional distinctions among fibroblast populations indicate their adaptation to specific anatomical regions and highlight their specialized roles in vascular support and regulation.

Although multiple subtypes of meningeal fibroblasts have been identified in mice, on the contrary, most human transcriptomic studies only classified CNS fibroblasts into perivascular and meningeal clusters due to the scarcity of cell numbers isolated from the CNS tissue. A recent study on human brain vasculature revealed that PVFs are more enriched in ECM-related molecules and functions than MFs, and they exhibit higher expression of type I collagen as the major component of fibrotic scar tissue [55]. Although both PVFs and MFs contribute to solute exchange of the cerebrospinal fluid flow, they might serve unique functions, with PVFs predominantly expressing ATP-binding cassette efflux pumps and MFs expressing solute carrier influx transporters [55]. Correspondingly, the fibroblast cluster with high solute carrier influx transporters expression was also identified in a study on human leptomeninges [56]. These findings were consistent with a study on mouse brain identifying fibroblast-like cell clusters that expressed either SLC transporters or ECM proteins, which respectively corresponded to MFs and PVFs [57]. Furthermore, it has become a growing area of interest to profile specifically the subpopulations of PVFs around the parenchymal vessels. In this case, it is crucial that the meninges are carefully removed to study purely PVFs in the brain tissue before sample lysis.

Meanwhile, several studies identified fibroblast subtypes, but their respective markers did not provide sufficient clues to distinguish them between MFs and PVFs. Vanlandewijck et al. [58] identified two distinct clusters of brain fibroblast-like cells in the mouse brain vasculature featuring different ECM expression profiles, with one cluster expressing higher level of *Col15a1*, and another cluster expressing higher level of a cell adhesion molecule *Itga8*. In the human brain samples, various clusters were identified across different studies, with one study distinguishing PVFs clusters with *DCN*/*APOD* or *PTGDS*/*ALDH1A2* expression as their marker genes, without investigating their functional differences [59]. Another study provided a more granular classification, which discovered *FBLN1* expressing PVFs as the most relevant to ECM organization, and another cluster labelled by *KCNMA1* with notable growth factors expression [60]. It is worth noting that PVFs markers identified in these studies overlapped with markers for fibroblasts of the meningeal origin, which indicated that these clusters may have distinct distribution patterns within the brain parenchyma. Details of CNS fibroblast clusters and markers are summarized here in Table 1.

**Table 1:** Clusters and markers of CNS fibroblasts and fibroblast-like cells

Cell Type	Definition	Cell clustering and markers
Meningeal Fibroblasts (MFs)	Fibroblasts located in the meninges, can be further classified as dural, arachnoid and pial fibroblasts	Human brain: Yang et al. [55]: MFs (solute carrier influx transporters), PVFs (ATP-binding cassette efflux pumps and extracellular matrix) Winkler et al. [59]: FB1 (*DCN*/*APOD*) or FB2(*PTGDS*/*ALDH1A2*) Garcia et al. [60]: Type I (*FBLN1*), II (*CEMIP*) and III (*KCNMA1*) Kearns et al. (leptomeningeal FB, 56): Fibro_1a (*SLC7A2*), Fibro_1b (*SLC7A2, TPRM3*), Fibro_2a (*LAMA2, TPRM3*) and Fibro_2b (*LAMA2*) Wang et al. (dual FB, 52): Three main clusters with proliferating, activation and ECM organization with immune regulation markers Adult mouse brain: Saunders et al. [57]: Seven clusters with selective expression of membrane transporters, pumps or extracellular matrix proteins Vanlandewijck et al. [58]: FB1 (*Col15a1*), FB2 (*Itga8*) Pietilä et al. [11]: PVFs (BFB1a, *Lama1*, *Col15a1*^high^), pial FB (BFB1b, *Lama1*, *Col15a1*^low^), arachnoid FB (BFB2-4, *Slc7a11*, *Ppp1r1a*, *Dpp4*), dural FB (BFB5, *Slc47a1*), PVFs on fenestrated vessels (BFB6, *Tcf21*) Developing brain: DeSisto et al. (Mouse, 44): At E14, pial FB (*S100a6*, *Col18a1*, and *Postn*), arachnoid FB (*Crabp2*, *Ogn*, *Aldh1a2*, and *Ptgds*), dural FB (*Fxyd5*, *Dkk2*, and *Alpl*), meningeal proliferating FB (*Top2a*, *Cenpa* and *Cenpf*) La Manno et al. (Mouse, 46): At E9, primitive fibroblast-like cells (*Mkx*, *Alx1*, *Alx3* and *Tbx18*) At E15, pial FB (*Col15a1*), arachnoid FB (*Slc22a2*), dural FB (*Ccn3*) and PVFs (*Tcf21*) Sun et al. (Human,^48^): Pial FB (*NGFR* and *ITIH5*), arachnoid FB (*CRABP2* and *ALCAM2*) and proliferating FB (*TOP2A* and *MKI67*)
Perivascular Fibroblasts (PVFs)	Fibroblasts found in the perivascular space around the penetrating arterioles, arteriole-capillary transition zones, and large venules	
Vascular leptomeningeal cells (VLMCs)	A cell cluster primarily identified from sequencing studies, equivalent to perivascular fibroblasts and fibroblasts in the leptomeninges (pia and arachnoid)	All in mouse CNS First introduced in Marques et al. [12]: *Pdgfra, Col1a1* Zeisel et al. [13]: VLMC1 (*Il33*) and VLMC2 (*Ptgds*) Rosenberg et al. [14]: *Slc6a13* + VLMCs and *Slc47a1* + VLMCs
*Glast* + perivascular cells	A group of stromal cells expressing glutamate aspartate transporter (*Glast*), including PVFs and a subset of pericytes	All in mouse CNS First introduced in Göritz et al. [15] and later elucidated by Holl et al. [87]: Cluster 1: type A pericytes (*Glast*/*Slc1a3*, *Kcnj8*, *Abcc9* and *Rgs5*) Cluster 2: PVFs (*Glast*/*Slc1a3, Col1a1*, Col3a1, *Lum*, *Dcn* and *Pdgfra*)

FB, fibroblasts; BFB, brain fibroblasts; E, embryonic day; P, postnatal day

Our understanding of CNS fibroblast functions during development and under physiological conditions continues to expand. Summarizing recent findings, PVFs and MFs share a close developmental relationship, yet exhibit distinct expression patterns of matrix components and solute transporters, suggesting specialized roles within the CNS. Notably, a subpopulation of PVFs characterized by *CEMIP* expression has a pseudo-time trajectory continuous with a subtype of pericytes, suggesting that these PVFs may represent a potential pool with pericyte progenitor-like properties, and potentially related to vascular structures maintenance [60, 61]. Another potential function of PVFs is contributing to and affecting cerebrospinal fluid flow by direct interaction and dynamic displacement in the perivascular compartments [43]. MFs, on the other hand, interact closely with the immune system and support neurogenesis by producing retinoic acid during CNS development [23, 62].

In neurological conditions, the stromal fibroblasts demonstrate functional complexity far beyond their well-characterized roles in ECM deposition and fibrotic process in the CNS. In both contusive and transection SCI, perivascular and pial fibroblasts form only the outer lesion layer, where they highly expressed type 4 collagen and laminin, and possibly related to angiogenesis and lipid transport. In contrast, fibroblasts originated from dura and arachnoid are located at the lesion center, with upregulation of type I collagen, fibronectin and cholesterol synthesis genes [32]. Their distinct collagen expression profiles reflect complex functional responses, though the regulatory programs driving these dynamics remain unknown. In human Alzheimer’s disease (AD) samples, the pathways of negative regulation of blood circulation, as well as dysregulated SMAD signaling and IFNγ pathways were found specifically in PVFs [55]. Besides, some novel AD-risk genes revealed by GWAS studies were enriched in the brain fibroblasts, including *FHL2*, *ECHDC3*, *ABCA1* for PVFs and *HESX1*, *IL34*, *APOE* for MFs. In Huntington’s disease, significant enrichment of Rap1, ErbB and HIF1 signaling pathways were found upregulated specifically in PVFs compared with controls, but not in other cells [60]. Indicated by other studies, these signaling pathways were highly related to fibroblast proliferation or ECM remodeling [63–65]. In amyotrophic lateral sclerosis (ALS), PVFs exhibit transcriptomic activation during presymptomatic stages across multiple mouse models, which coincides with pathological hallmarks of enlarged PVS and aberrant accumulation of extracellular matrix proteins and chemokines in human ALS tissues [66].

In conclusion, many of these findings require functional validation, and it is important to consider that the characteristics and behaviors of CNS fibroblasts may vary between human and mouse models, especially under different pathological conditions. These differences could influence how findings from animal studies translate into human diseases. Recently, the enlarged PVS gained growing interest in the association with cerebrovascular diseases and neurodegenerative diseases, which also highlighted the potential role of PVFs in pathophysiology [67, 68]. Although fibroblasts represent one of the minor cell populations of CNS, they show multiple functions and are linked to many neurological diseases, suggesting the need for further exploration of their roles in health and diseases. Meanwhile, since fibroblasts are one of the rare cell types in the CNS in healthy individuals, it is necessary to pay extra attention to the quality of sequencing studies that might reveal the subtle expression changes [69]. Being transparent of sample information, quality control procedures and key steps in analysis such as clustering approaches, as well as proper experimental validation, can facilitate independent quality assessment and re‑analysis by the whole research community.

## VLMCs and their connections to CNS fibroblasts

The term VLMCs was introduced in one of the first single-cell sequencing studies that analyzed *Pdgfra*-GFP-positive cells from mouse brains, which intended to label all cells of the oligodendrocyte lineage [12]. Apart from *Pdgfra* + oligodendrocyte precursor cells (OPCs), a distinct cluster of cells characterized by *Pdgfra* and *Col1a2* expression but lacking *Sox10*, *Sox6* and *Cspg4* markers were identified, and further histological analysis confirmed that these cells are located in perivascular and meningeal regions [12]. This suggested that this VLMC cluster, initially used to annotate *Pdgfra* + cells that were not OPCs in single-cell studies, are CNS fibroblasts. The overlapping *Pdgfra* expression highlights the need to use additional markers to differentiate VLMCs from oligodendrocyte lineage cells, as OPC development and migration is tightly related to vascular structures [70]. Since then, the VLMCs designation has been predominantly employed in several scRNAseq transcriptomic studies aimed to broadly map the brain cell types including the projects led by the Allen Institute and others [57, 71, 72]. A subsequent study described two subtypes of VLMCs in the adolescent mouse nervous system, including one with high expression of proinflammatory cytokine *Il-33*, and another leptomeninges-related subtype with high expression of prostaglandin D2 synthase encoded by the *Ptgds* gene [13]. Comparable results were observed in the developing mouse CNS, where two VLMC clusters, characterized by high expression of either *Slc6a13* or *Slc47a1*, were identified [14]. Notably, *Scl47a1* gene was also identified as a marker for MFs in another study [55]. Even though the ECM related expression profiles of VLMCs populations were not thoroughly explored in these studies, they were at least clearly distinguished from other vascular cell types, including pericytes. Table 1 also summarizes the details regarding VLMC clusters and their markers.

The combination of scRNA-seq and high-resolution spatial transcriptomics facilitated the characterization of cell–cell interactions and functions in healthy, biological aging and disease contexts. Pial-specific and parenchymal VLMCs display distinct spatial distributions and differential expression of extracellular matrix components and transporter subtypes in the mouse brain [72, 73]. Notably, the pial VLMCs population colocalizes with the interlaminar astrocytes located at layer I in the brain parenchyma, which indicates potential glia–vasculature crosstalk. Certain VLMC clusters show close spatial association with tanycytic and choroid plexus ependymal cells, suggesting their involvement in regulating cerebrospinal fluid function [72]. Taken together, results from these studies were consistent with anatomical distribution of CNS fibroblasts in meninges, PVS and choroid plexus [2]. In the aging brain, VLMCs and other vascular cells tend to stay more proximal to each other, likely indicating maturation or remodeling of neurovasculature [74]. Aging was also found to impact VLMCs at the transcriptomic level, with one VLMC cluster exhibiting decreased expression of genes involved in extracellular matrix organization and collagen synthesis [75]. Interestingly, another VLMCs cluster and several non-neuronal cell types were related with reduced neuronal and synaptic functions, a relationship that remains to be fully understood [75]. VLMCs have been suggested to influence astrocyte responses during LPS‑induced neuroinflammation, as spatial transcriptomic analysis indicated the correlation of astrocytes activation states and their proximity to VLMCs [74].

These studies allowed VLMCs characterization with unprecedented depth and resolution, and revealed that VLMCs and CNS fibroblasts display highly similar subtypes, transcriptomic profiles, spatial distributions and functions within the brain. Therefore, the terms “VLMCs” and “CNS fibroblasts” appear in parallel in the literature, leading to a misperception that these are separate cell types. These terms refer to the same cell type, although considering their nuanced phenotypes in the brain cortex, meninges and specialized structures like choroid plexus, and future studies will use categories to differentiate these subtypes. The evolving nomenclature for these cells reflects the ongoing advancement of our understanding of these rare cells.

## Beyond type A pericytes: revealing the heterogeneity of *Glast* + perivascular cells

From a traditional perspective, pericytes are typically present on capillaries, and together with the smooth muscle cells (SMCs) on arteries or veins they form the arteriovenous continuum of mural cells on vessels [76, 77]. An early study demonstrated an acute response of pericytes to traumatic brain injury by migrating into the lesion away from their original perivascular location [78]. This was subsequently investigated through a series of studies employing transgenic Cre-line mouse models driven by what were at the time considered pericyte-specific promoters, such as *Pdgfrb* or *Cspg4* (NG2). In the contusive SCI models, *Cspg4* expressing pericytes were only found at the outer layer of the lesion while *Col1a1* expressing fibroblasts located mostly at the core, and these cells rarely overlapped in the lesion [7]. The claim of pericyte contribution to scar formation was further reinforced in another study which showed that proliferating *Cspg4* positive cells were in the glial scarring region, and they supported post-injury angiogenesis into the lesion site and regulated fibrotic scar formation [79]. However, single-cell RNA sequencing studies showed that the *Pdgfrb* or *Cspg4* expression is not restricted to pericytes [58]. Therefore, mouse models only labeling *Pdgfrb*-expressing cells are not suitable for studying pericyte functions and behaviors, since an overlap of the same markers with SMCs and fibroblasts confounded the interpretation of results. Similarly, *Cspg4* proteoglycan expression is not exclusive to pericytes, but also observed in oligodendrocyte progenitor cells, astrocytes, myeloid macrophages, and microglia, making it necessary to combine multiple markers when studying NG2-positive pericytes [80–82]. A specific pericyte subpopulation with a distinct abluminal location to typical pericytes was identified in the basement membrane when studying SCI in the *Glast*-CreER^T2^ mouse strain crossbred with reporter mice [15]. This pericyte subtype, named “type A pericytes”, demonstrates typical pericyte markers PDGFRβ and CD13 but not desmin. Upon SCI, “type A pericytes” are highly proliferative and differentiate into fibrotic scar-forming cells expressing alpha smooth muscle actin and fibronectin. Ultrastructural analysis showed that only “type A pericytes” detach from the vasculature after injuries and deposit various matrix proteins around the injury site. Later, similar response of proliferation, migration and ECM secretion profiles by the “type A pericytes” were proved to be consistent across other CNS lesions and neurological disease models, including traumatic injuries, ischemic stroke, experimental autoimmune encephalomyelitis and glioma, suggesting the common features with minor difference in the patterns of CNS fibrosis [16]. Although inhibiting the proliferation of “type A pericytes” impairs the formation of the fibrotic scar after SCI, this intervention has been shown to significantly enhance the regeneration of serotonergic neurons and the corticospinal tract [15, 83]. This suggests that modulating CNS fibrosis can serve as potential therapies in many traumatic neurological conditions. While *Glast*, also known as the *Slc1a3* gene, has been used as a marker for pericytes, its application presents some challenges. Subsequent single cell sequencing studies showed that the expression of *Glast*/*Slc1a3* is present in other brain cell types including fibroblasts and astrocytes [58]. *Glast*-CreER^T2^ mice were previously applied in astrocyte studies, but the efficiency of *Glast*-CreER^T2^ recombination varies across different brain regions and by whether it is a knock-in or transgenic mouse line, likely reflecting differences in both *Cre* activity and *Glast* expression levels among astrocytes and other cells [84–86]. Several transcriptomic databases indicated diversifying expression levels of *Glast* in astrocytes, pericytes, PVFs and MFs [11, 55, 57, 58]. Elevated expression of the *Slc1a3* gene was observed in a specific cluster of pericytes characterized by their enrichment of transmembrane transporters, and in another study, a specific pericyte cluster situated near arterioles [55, 60]. Therefore, *Glast*/*Slc1a3* expression on vascular cells represents heterogeneous clusters that may contribute to various aspects of CNS physiology and pathology. A recent study provided insights into diversity of *Glast* + perivascular cells, and revealed that *Glast* expression characterized a cluster of pericytes and most of the PVFs (Table 1) [87]. Therein both *Glast*-CreER^T2^ and *Col1a1*-CreER^T2^ transgenic mice label overlapping populations of PVFs in penetrating arterioles and venules, and pericytes were exclusively marked by *Glast* expression on smaller arterioles and capillaries. Correspondingly, *Col1a1* + */Glast* + PVFs are more prominently involved in scar formation within white matter where most vessels of large diameter are located, whereas *Col1a1-/Glast* + pericytes primarily contribute to gray matter fibrosis following neuroinflammation and SCI, depicting a region-specific pattern [87]. Nonetheless, it is worth noting that the recombination efficiency for the *Col1a1-*CreER^T2^ line varied and was the lowest for all *Col1a1* expressing cells in grey matter, which might cause underestimation of PVFs contribution to tissue fibrosis in this region [87]. Following injury, fibroblasts across multiple organs become activated and transition into a specialized state termed myofibroblasts, with remarkable matrix protein and cytokine production and contractile capabilities [88, 89]. The transformation of fibroblasts to myofibroblasts was reported in SCI and stoke models [7, 90]. Upon CNS injury, gene expression pseudo-time analysis on *Glast* + cells revealed the convergent transformation from both a distinct subpopulation of "type A pericytes" and perivascular fibroblasts through myofibroblast cluster and finally to the activated fibroblasts [87]. Although the meninges were removed in this study, leaving certain aspects unresolved, the comprehensive understanding of these *Glast* + stromal cells clarified the roles of both PVFs and a pericyte cluster in CNS fibrosis, and opened the possibility of targeting specific cellular states during their transitional processes.

Nonetheless, the involvement of *Glast* + pericytes in CNS fibrosis after injury remains under debate, as another report indicated the lack of pericyte markers in the proliferating cells after injury in mouse and monkey models [32]. Tracing pericyte contribution to CNS scarring using the *Atp13a5*-CreER^T2^ mice revealed no contribution of Atp13a5 + pericyte lineage cells to the stroke lesion [31]. Another study followed the *Hic1* expressing stromal progenitor populations in the CNS after stroke and identified activation and proliferation of pericytes and perivascular fibroblasts [91]. Interestingly, within the *Hic1* + cell populations, *Glast* expressions were only enriched in PVFs but not pericytes, suggesting the heterogeneity within pericytes. One general challenge arises in characterizing cell status upon cellular transitional stage after activation in injuries because pericytes rapidly lose *Atp13a5* and other pericyte related gene expression [87]. To resolve this question, further studies using *Atp13a5-*CreER^T2^ mouse model that can recombine pericyte exclusively could address pericytes behavior under other neurological conditions, including neuroinflammation and spinal cord injuries [31, 92]. In the future, comprehensive analysis on how *Glast* + cell transformation is regulated by different transcription factors, and what their regional differences are in the CNS, would be necessary to understand the heterogeneity of *Glast* + perivascular cells.

Taken together, these findings highlighted that *Glast* expression on perivascular cells marks both "type A pericytes" and PVFs, which represent distinct subsets of stromal cells contributing to CNS fibrosis. This underscores the close functional and potentially developmental relationship between pericytes and CNS fibroblasts, and emphasizes the need for precise nomenclature to distinguish these overlapping stromal cell populations in future studies.

## Conclusion

Since the last century, research on the cellular origins of CNS fibrosis has progressed rapidly. Advances in genetic lineage tracing and single-cell transcriptomics uncovered the heterogeneity and plasticity of CNS fibroblasts and other stromal cells but also highlighted significant challenges in cell identification and nomenclature. This review presents an overview of the current understanding of CNS fibroblasts, including MFs and PVFs that are anatomically segregated but related in functions and transcriptomics. They were occasionally referred collectively to as VLMCs in some studies. Meanwhile, PVFs and a cluster of pericytes were identified as *Glast* + perivascular cells that display fibrotic capacity during CNS injury and repair (Fig. 1). Nonetheless, even with ongoing tissue-derived scRNA/snRNA-seq studies that help to understand cell heterogeneity, a comprehensive characterization of cell states and identities will rely on inclusion of CNS fibroblasts into respective *in vitro* models. While generation of fibroblasts with CNS characteristics would provide scalable phenotypic indications, it may be challenging for them to fully recapitulate features of native CNS fibroblasts. Another challenge of this field is to characterize the cells during the transitional stage in various neurological conditions, due to the lack of specific cellular markers. Meanwhile, the development and application of multiple lineage-tracing tools is crucial for determining cell types that are involved in the complex fibrotic process, and the interpretation of the results should account for the biological background of the mouse strain, including whether it is a knock-in or transgenic line. We believe that the parallel comparison of CNS stromal cells will improve our understanding of their origins and functions and help resolve ongoing controversies regarding their classification and relationships within the research community. Moving forward, integrative multi-omics approaches will be essential to clarify the roles of brain fibroblasts and fibroblast-like cells in CNS health and diseases.

## Data Availability

No datasets were generated or analysed during the current study.

## Abbreviations

AD: Alzheimer’s disease
ALS: Amyotrophic lateral sclerosis
BFB: Brain fibroblasts
CNS: Central nervous system
E: Embryonic day
ECM: Extracellular matrix
MFs: Meningeal fibroblasts
P: Postnatal day
PVFs: Perivascular fibroblasts
PVS: Perivascular space
SCI: Spinal cord injury
SMCs: Smooth muscle cells
VLMCs: Vascular leptomeningeal cells
