# Correction: From origins to nomenclature: illuminating the landscape of CNS fibroblasts and fibroblast-like cells

**DOI:** 10.1186/s40478-026-02338-3

**Published:** 2026-06-01

**Authors:** Jianing Lin, Kuo Liao, Sebastian A. Lewandowski

**Affiliations:** Department of Clinical Neuroscience, Centre for Molecular Medicine, Karolinska Institutet, Karolinska Hospital, Stockholm, Sweden

**Correction to: Acta Neuropathologica Communications (2026) 14:53** 10.1186/s40478-026-02236-8

There is a duplication of a paragraph under the CNS fibroblast subtypes and their proposed functions section of this article [1], where the same text appears twice consecutively. As a result, the duplicated paragraph has been deleted.

The following paragraph was the one appearing twice:

“Meanwhile, several studies identified fibroblast subtypes, but their respective markers did not provide sufficient clues to distinguish them between MFs and PVFs. Vanlandewijck et al. [58] identified two distinct clusters of brain fibroblast-like cells in the mouse brain vasculature featuring different ECM expression profiles, with one cluster expressing higher level of Col15a1, and another cluster expressing higher level of a cell adhesion molecule Itga8. In the human brain samples, various clusters were identified across different studies, with one study distinguishing PVFs clusters with DCN/APOD or PTGDS/ALDH1A2 expression as their marker genes, without investigating their functional differences [59]. Another study provided a more granular classification, which discovered FBLN1 expressing PVFs as the most relevant to ECM organization, and another cluster labelled by KCNMA1 with notable growth factors expression [60]. It is worth noting that PVFs markers identified in these studies overlapped with markers for fibroblasts of the meningeal origin, which indicated that these clusters may have distinct distribution patterns within the brain parenchyma. Details of CNS fibroblast clusters and markers are summarized here in Table 1.”

The original article has been corrected.
